# A high prevalence of cumulative trauma disorders in Iranian instrumentalists

**DOI:** 10.1186/1471-2474-5-35

**Published:** 2004-10-14

**Authors:** Shahram Sadeghi, Behrooz Kazemi, Seyed Mostafa Jazayeri Shooshtari, Ali Bidari, Peyman Jafari

**Affiliations:** 1 Pain research group, academic centre for education, culture and research, Iran medical science branch. No 31, Karimkhan zand Avenue, shahid hosseini alley, multidisciplinary pain clinic. Tehran, Iran; 2 Department of physical medicine and rehabilitation, Shiraz medical school, Shiraz, Iran; 3 Emergency department, Hazrat-e-rasoul medical complex. Iran university of medical sciences, Tehran, Iran; 4 department of biostatistics, Shiraz medical school, Shiraz, Iran

## Abstract

**Background:**

Cumulative trauma disorders (CTDs) are common in musicians and their prevalence has been the subject of a number of studies in most western countries. Such studies are scarce in developing countries despite the possibility that CTDs may have a different prevalence in these countries, especially when considering traditional musical instruments and different methods of playing. Although not formally studied before, according to our experience the prevalence of CTDs seemed to be high among Iranian instrumentalists.

We proposed this study to determine the prevalence of CTDs in amateur music students playing one of the two traditional Iranian instruments: Daf and Setar.

**Methods:**

In a prospective cross sectional study, we interviewed and examined the students of three music training centers in Iran. Seventy eight instrumentalists, who were playing Daf or Setar and twelve students who had not started playing yet were regarded as case and control groups respectively. Some of them also underwent electrodiagnostic studies.

**Results:**

Forty-seven percent (17 of 36) of the Setar players and 57% (24 of 42) of the Daf players and fifty-three percent (41 of 78) of the instrumentalists as a whole had CTDs. None of them had carpal tunnel syndrome.

**Conclusions:**

Our study revealed that the prevalence of CTDs in Iranian instrumentalists was unusually high. In addition to age, other variables may be contributory. This needs to be further studied.

## Background

Cumulative trauma disorders, also called repetitive stress injuries, overuse syndromes or repetitive motion injuries [1-3] are common in musicians [4,5] and are caused by repetitive motions. Nerve entrapments, stress fractures, tendonitis, bursitis and muscle strains have been labeled in this category [1,5].

To date no study has been performed about the prevalence of cumulative trauma disorders (CTDs) in players of Iranian instruments. According to our experience, it seemed to be unusually high when compared with related prevalence in nonprofessional players of classical instruments as reported by Fry [4].

This study was performed to determine the prevalence of cumulative trauma disorders in amateur music students playing two traditional Iranian instruments: Daf and Setar.

**Figure 1:**
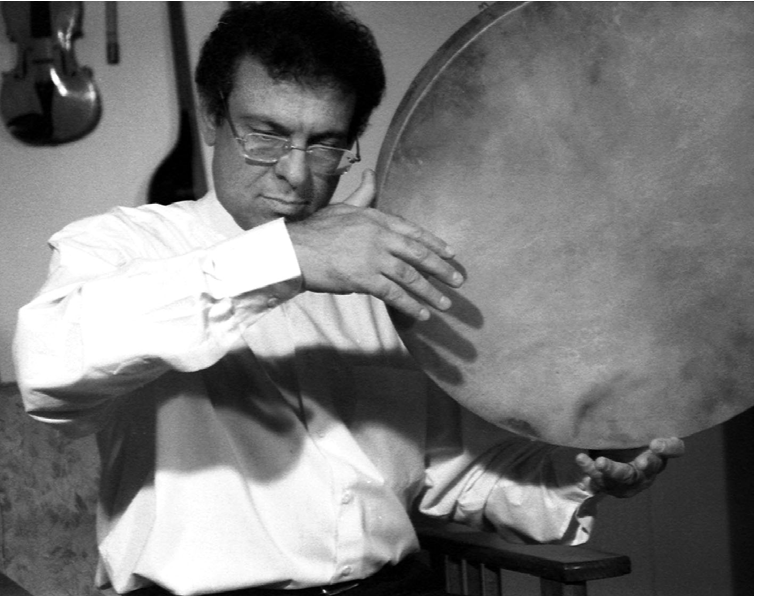
Bijan Kamkar (one of the most famous Iranian Daf players/teachers) showing the playing technique for Daf. With thanks to Hana Kamkar (photographer).

Daf is a percussion musical instrument that has a circular wooden frame covered with goat skin with or without metal discs around its edge. To play Daf the player shakes it and hits it with both hands (fig. 1).

**Figure 2:**
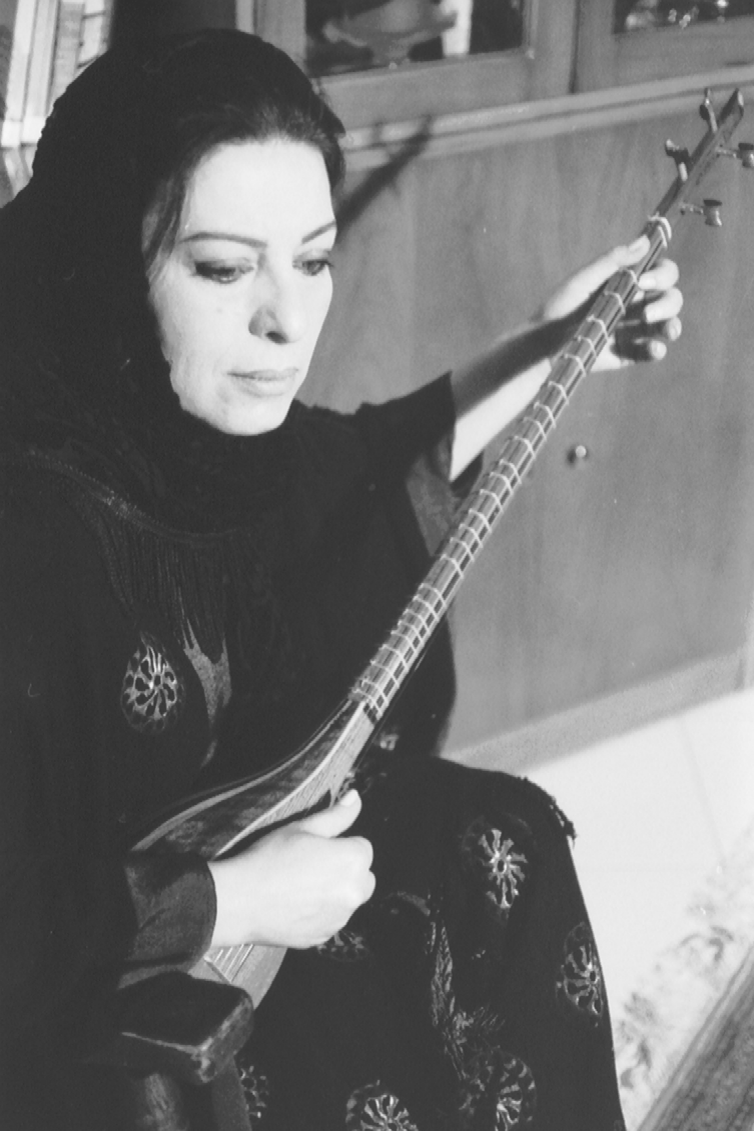
Ghashang Kamkar (Setar player/teacher) showing the playing technique for Setar.

Setar is a string musical instrument that has 4 strings. It is played by the index of right hand (fig. 2).

In comparison to classic musical instruments, Setar resembles the Guitar, but Daf doesn't have any similar equivalent.

## Methods

In a prospective cross sectional study, we interviewed and examined the students of three music training centers, numbering 94. Twelve students who were at their first sessions and hadn't begun to play were selected as control group.

Age, sex and duration of playing (date of starting and daily playing time) as well as vocational and avocational risk factors for developing CTDs were recorded after a direct interview. Then the students were referred to a physician who did not know whether the student belonged to the case or control group. He then evaluated their upper limbs and necks. Specific attention was paid to pain, paresthesia, sensory changes, tenderness, range of motion, muscle power and muscle stretch reflexes. In addition, Phalen, Tinel and carpal compression tests [6] were performed to detect the presence of carpal tunnel syndrome (CTS); the most common neuropathy reported in instrumentalists [5].

Since the standard diagnostic test for CTS is electrodiagnostic study [7], all of the students were asked to attend our center for electrodiagnostic studies. In all of the participants, antidromic median sensory nerve action potential (SNAP) was obtained from the third digit at both 7 and 14 cm. Then the split times and amplitudes were compared. Also distal latency for the motor median nerve was obtained. We also compared the wrist versus midpalm compound muscle action potential (CMAP) amplitudes [8-10]. Electromyographic investigation was not performed.

The data were analyzed by SPSS software using Chi square and Fisher's exact tests.

## Results

Ninety four students were included in this study. Four students were excluded from the study, two because of a history of musculoskeletal pain before attending the music center, one because of playing two instruments and one, serving as a typist.

Twelve of the students who had not started to play were assigned to the control group and the remaining 78 students; 42 in Daf and 36 in Setar groups; were considered as case group (table 1).

Mean age of students in the case group was 21.2 years (SD: 3.8) including 47 females and 31 males. Mean duration of instrument playing in this group was 7.9 months (SD: 5.4).

Mean age of students in control group was 25.2 years (SD: 9.2). This group consisted of 9 females and 3 males.

Mean Duration of daily playing in Setar students was more than Daf students (1.6 Vs 1.5 hours) which was not statistically significant (P value = 0.8).

Mean duration of daily playing in male and female students was 1.8 hours and 1.4 hours respectively. Which was not statistically significant (P value = 0.64).

Forty-one students in case group (53% of the total of 78) had musculoskeletal pain and there was a significant correlation between playing Daf and Setar and development of musculoskeletal symptoms.

The prevalence of pain among females was twice as much as males but the difference was not statistically significant (P value = 0.12) (table 2).

The prevalence of musculoskeletal pain in Daf players was more than Setar players (57%vs 47%, P value = 0.38); again, this difference was not significant (table 2).

Regarding the location of pain, hand was the most common site; it was painful in 65% of cases (table 3).

Twenty six students, all from the case group attended our electrodiagnostic center, none of them had carpal tunnel syndrome.

None of the students in control group had problems in their exams and none of them attended for electrodiagnostic studies.

## Discussion

A large number of amateur Daf and Setar players with a history of playing of less than 1 year (7.9 months) and almost 1.5 hours a day had musculoskeletal pain (that is considered a form of CTDs). The prevalence of pain in this group was much greater than students in tertiary music schools who train for some years for 6 hours a day (53% vs. 9.3–21%, respectively) and almost equals professional orchestra players (73–75%) [5].

In a group of instrumentalists (Guitarists, Harpists, Pianists etc.) Bejjani et al found a 77.5% prevalence of upper extremity disorders serious enough to impair the performance or to cause the musician to stop playing at least temporarily [11]. similarly, it is possible that some of the students also had quit playing before they had chance to enter our study (case selection bias) this might have caused an underestimation of the prevalence of the CTDs observed in this study. So it may be reasonably concluded that 53% is the minimum prevalence of CTDs in the studied group.

How can we explain this high prevalence?

The most important cause of CTDs is repetitive motions and in fact multiplication of duration and intensity of exercise [4]. Since both the duration and the intensity of exercise in these players were much less than that of professional music students or music trainees, other factors should be considered.

According to Fry [4], other important factors that predispose to CTDs are genetics and student technique. Since the students were taught in certified centers and the music teachers were satisfied with the students' techniques, we assumed that playing method was not of primary concern.

The particular instrument has been shown to be a risk factor for developing CTDs [5,14]. On the other hand, Setar is not heavier than guitar, nor does its playing need awkward positions, so the instrument in itself may not explain this high prevalence.

Another risk factor is age [12]. It has been shown that adults who start playing, may be more vulnerable to developing CTDs. The extent to which this may have affected the results of our study is not clear so we additionally proposed that the studied group might have been inherently susceptible to develop CTDs because of some genetic factors such as joint hypermobility.

This hypothesis can explain, at least in part, the wide range (9–49%) [5-12] of the prevalence of CTDs in music students by different studies. However, genetic analyses and larger studies are needed for validating this hypothesis.

As mentioned, we did not find any case of symptomatic carpal tunnel syndrome or nerve conduction abnormalities suggesting subclinical median neuropathy at the wrist, implying that CTS is a more advanced form of cumulative trauma disorders when compared to musculotendinous unit CTDs.

Hand was the most common painful site in this study (65%); a finding which is in concert with other published studies (41–54 %)[4,11].

There are two other findings in the current study left to be explained:

First: Daf is played being held using both hands but Setar is being held like Guitar. So it can be postulated that playing Daf is more harmful than Setar and we expected more CTDs in Daf players. Although, the prevalence of CTDs was higher in Daf players, the difference was not significant (p value = 0.38). A significant difference may be found with a large scale study.

Second: it has been known that CTDs are more common in females [5,12,13]. In our study, we also found a higher occurrence of CTDs among females but the study failed to reveal a statistically significant difference (p value = 0.12), perhaps because of small sample size.

## Conclusions

Our study revealed that the prevalence of CTDs in Iranian instrumentalists was abnormally high. This is an unusual finding that can't be fully explained by the difference in the instruments (classical versus traditional), playing method or intensity of the exercise. Other susceptibility factors such as age at the starting of playing or genetic predisposition may be contributory. Larger studies focusing on individual characteristics and genetic analyses are needed to delineate other important factors.

## Competing interests

The authors declare that they have no competing interests.

## Abbreviations

CTD: cumulative trauma disorders

CTS: carpal tunnel syndrome

## Authors' contributions

SS: suggesting the proposal, examining the volunteers, writing the paper.

BK & SMJS: examining the volunteers

AB: great help in writing the paper and statistical analysis

PJ: statistical analysis

## Pre-publication history

The pre-publication history for this paper can be accessed here:



## Figures and Tables

**Table 1:** Sex distribution of students

Instrument/Gender	male	female	total
Daf	15	27	42
Setar	16	20	36

**Table 2:** cumulative trauma disorders regarding instrument and gender

Instrument/Gender	female	male	Total
Daf	19	5	24
Setar	9	8	17

**Table 3:** Location of pain and its relation to different instruments (some students have had more than one painful area)

Instrument/pain location	hand	forearm	arm	Coracoid process
Daf	14	8	4	8
Setar	13	4	1	1
